# LncRNA RP11-59J16.2 aggravates apoptosis and increases tau phosphorylation by targeting MCM2 in AD

**DOI:** 10.3389/fgene.2022.824495

**Published:** 2022-08-26

**Authors:** Fulin Guan, Qichang Gao, Xinghua Dai, Lei Li, Rui Bao, Jiaao Gu

**Affiliations:** ^1^ Department of Neurology, The First Affiliated Hospital of Harbin Medical University, Harbin, China; ^2^ The First Affiliated Hospital of Harbin Medical University, Harbin, China; ^3^ Haiyuan Hospital of Heilongjiang, Harbin, China; ^4^ Integrated Chinese and Western Medicine Department, The First Affiliated Hospital of Harbin Medical University, Harbin, China; ^5^ Heilongjiang University of Chinese Medicine, Harbin, China

**Keywords:** Alzheimer disease, microarray analysis, RP11-59J16.2, MCM2, apoptosis, P-tau

## Abstract

Alzheimer’s disease (AD) is a degenerative disease of central nervous system with unclear pathogenesis, accounting for 60%–70% of dementia cases. Long noncoding RNAs (LncRNAs) play an important function in the development of AD. This study aims to explore the role of differentially expressed lncRNAs in AD patients’ serum in the pathogenesis of AD. Microarray analysis was performed in the serum of AD patients and healthy controls to establish lncRNAs and mRNAs expression profiles. GO analysis and KEGG pathway analysis revealed that G_1_/S transition of mitotic cell cycle might be involved in the development of AD. The result showed that RP11-59J16.2 was up-regulated and MCM2 was down-regulated in serum of AD patients. SH-SY5Y cells were treated with Aβ 1–42 to establish AD cell model. Dual luciferase reporter gene analysis verified that RP11-59J16.2 could directly interact with 3′UTR of MCM2 and further regulate the expression of MCM2. Inhibition of RP11-59J16.2 or overexpression of MCM2, CCK-8 assay and Annexin V FITC/PI apoptosis assay kit results showed that RP11-59J16.2 could reduce cell viability, aggravate apoptosis and increase Tau phosphorylation in AD cell model by inhibiting MCM2. In short, our study revealed a novel lncRNA RP11-59J16.2 that could promote neuronal apoptosis and increase Tau phosphorylation by regulating MCM2 in AD model, and indicated that lncRNA RP11-59J16.2 might be a potential target molecule for AD development.

## Introduction

Alzheimer’s disease (AD) is most common cause of dementia in aging population. Involved with a complex neurodegenerative pathogenesis, the accumulation of β-amyloid and intracellular neurofibrillary tangles are the main pathological hallmarks of AD (Galvão et al., 2019). By 2050, there will be more than 100 million AD patients over the world (Tarditi et al., 2009). AD is life-threatening and constitutes a considerable portion of the medical and care burden to families and society. Due to the unclear etiology and complex pathogenesis, there is no way to reverse the disease process (Toffa et al., 2019). Therefore, it is important to study the potential mechanism of AD development.

It was reported that non-coding RNA (ncRNA) such as lncRNA and microRNA plays an important role in cell survival (Brosnan and Voinnet, 2009; Guttman et al., 2009). Long non-coding RNAs (lncRNAs) are non-coding RNAs with more than 200 nucleotide sequences. Their regulatory role in tumors has been explored worldwide (Shi et al., 2013; Chen et al., 2018). LncRNAs regulate cell progression through targeting miRNAs or mRNA (Gutschner and Diederichs, 2012; Paraskevopoulou and Hatzigeorgiou, 2016). More and more evidence shows that lncRNAs can be used as important molecular markers involved in gene regulation and cell progression (Sun et al., 2020). Mini-chromosome maintenance complex component 2 (MCM2) gene is a major gene affecting the stability of micro mitotic chromosomes Ⅲ, and MCM2 protein is a family member that initiates DNA replication and maintains progression in the cell cycle (Gottesman et al., 2002). Human MCM2 was first reported in 1998 (Randell et al., 2006). MCM2 is located on chromosome 3q21.3 and comprises 17 exons (Kucherlapati, 2018). According to recent reports (Blow and Dutta, 2005), MCM2 was verified as a cell marker of cell proliferation which indicated that MCM2 could repair neuronal injury in AD through promoting neuronal injury. Previous study (Bonda et al., 2009) showed that phosphorylated MCM2 was strikingly associated with the characteristic NFT in AD and its localization in the cytoplasm of neurons suggests its effect on resultant cell cycle stasis and consequent neuronal degeneration.

We studied the expression profiles of lncRNAs and mRNAs in serum of AD patients, and found that the expression of lncRNA RP11-59J16.2 and MCM2 in AD patients has significant differences compared with healthy control. However, the role of RP11-59J16.2/MCM2 in the development of AD was unclear. In this study, the effect of RP11-59J16.2/MCM2 on the development of AD would be investigated to explore the molecular mechanism of the novel lncRNA RP11-59J16.2 on regulation of Aβ-induced neuronal damage in the AD cell model by targeting MCM2, and to provide a potential target and theoretical basis for the treatment of AD.

## Materials and methods

### Ethics statement

The patients included in the present study provided written informed consent. The study was approved by the ethics committee of Harbin Medical University.

### Sample collection

AD patients aged 65–75 who met the diagnostic criteria for AD were selected from Neurology Department in the first affiliated hospital of Harbin Medical University. Peripheral blood samples of AD patients and healthy control with the same age group were collected. Inclusion criteria for AD patients were as follows.

For the AD patients: (1) Participants aged 65–75  years were recruited; (2) It conforms to the National Institute of Neurological and Communicative Disorders and Stroke/Alzheimer’s disease Criteria (NINCDS-ADRDA) for diagnosis of probable Alzheimer disease; (3) Subjects or their caregivers voluntarily participate in the study and sign the informed consent. Subjects were excluded if they had a history of head trauma. Exclusion criteria: (1) Age <65 years. (2) Other dementia or other genetic history in the family. (3) Patients with significant depressive symptoms with Hamilton Depression Scale (HAMD) score ≥15 or patients with vascular dementia with Hachinski Ischemia Scale (>) score 7. (4) Patients who suffer from other serious medical conditions.

For the healthy control: (1) Healthy subjects aged 65–75 years old, same sex as matched AD patients; (2) No major systemic diseases in heart, liver and kidney disease.

### Differential expression profile of mRNA and long noncoding RNA in whole blood of alzheimer’s disease patients

2 ml whole peripheral blood was taken from 3 AD patients and 3 healthy people respectively. Total RNA was extracted. Agilent ND-1000 was used to detect RNA degradation and RNA concentration (Aranda et al., 2009). Samples were labeled with Arraystar RNA Flash Labeling Kit (Shi and Shang, 2016). After hybridizing, incubating and washing, the chip was scanned using Agilent RNA Microarray Scanner. Agilent Feature Extraction software (V11.0.1.1) was used to collect the chip probe signal value (Zahurak et al., 2007). Agilent GeneSpring GX V12.1 software was used for chip quantile standardization. The microarray data have been uploaded in the National Center for Biotechnology Information Gene Expression Omnibus (GEO, https://www.ncbi.nlm.nih.gov/geo/) [GSE 182910: GEO].

### Differential expression analysis

Comparing gene profiles of AD and healthy control, fold change (FC, the ratio of the group average) in each lncRNA was calculated. *t*-test was used to estimate the statistical significance. LncRNAs with FC ≥ 2.0 and *p* value ≤ 0.05 was selected as the differentially expressed genes. Microsoft Excel’s Data/Sort & Filter functionalities were used to filter the analysis output, and the differentially expressed LncRNAs was sorted according to the fold change and *p* value.

### Gene ontology annotation and encyclopedia of genes and genomes pathway enrichment analyses of the differentially expressed genes

Gene Ontology (GO), including biological processes (BPs), cellular components (CCs), molecular functions (MFs) and Kyoto Encyclopedia of Genes and Genomes (KEGG) pathways enrichment analyses of differentially expressed genes (DEGs) were performed using the Database for Annotation, Visualization and Integrated Discovery (*p* < 0.05 as the criteria for enrichment significance) (Chen et al., 2017).

### Protein-protein interaction network construction

In order to further explore the interaction of different target genes and the molecular mechanism of AD, we used STRING (https://string-db.org/) to analyze and construct a PPI network. Then the interaction network and the top 50 hub genes were visualized by cytoHubba in Cytoscape. The nodes in the network were represented as target genes, and the lines between two nodes were denoted interactions.

### Cell culture

Human neuroblastoma cell line (SH-SY5Y) was purchased from Beina Cell Bank and cultured in DMEM/F12 complete medium containing 10% fetal bovine serum and 1% antibiotics. The cells were cultured in an incubator containing 5% CO_2_, 95% humidity and 37°C. SH-SY5Y was treated with different concentrations (0, 5, 10, or 20 µM) of Aβ for 24 h or 10 µM of Aβ for different times (0, 12, 24, or 48 h) to establish *in vitro* AD cell model. si-RP11-59J16.2, si-RNA negative control (si-NC), si-MCM2, pcDNA-MCM2, pcDNA-NC were all purchased from Sangon Biotech (Shanghai) Co., Ltd. The antibody was purchased from Abcam. CCK-8 cell viability detection kit and Annexin V-FITC/PI Cell Apoptosis Assay kit were purchased from BEENbio Biotechnology.

### Real-time quantitative PCR

Trizol was used to extract total RNA from each group, and cDNA was obtained by reverse transcription according to the instructions. The reverse transcription system was as follows: RNase Free dH2O: 14µl, 5X all in one reverse transcription mix: 2µl, RNA: 4µl, 42°C for 15min and 85°C for 5s for reverse transcription. Real-time quantitative PCR detection (reaction system was 20 µl). RT-qPCR system was as follows: 2*SYBE green qPCR mix: 10µl, cDNA template: 20ng, forward primer (5 µM): 1µl, Reverse primer (5 µM): 1µl, ddH2O: add to 20µl, qPCR program: 95°C hold for 5 min, and followed 40 cycles as 95°C 30s and 60°C for 45s. GAPDH was used as the reference gene, mRNA expression abundance in the treated cells in each group was calculated as △Ct (△Ct = Ct value of target gene—Ct value of internal reference gene). Target gene primer sequence: MCM2 (For 5′-AGA​GGA​TCG​TGG​TAC​TGC​TAT​GGC-3′, Rev 5′-TTA​TGG​ATG​GCA​TAG​GGC​CTC​AGA-3′), RP11-59J16.2 (For 5′-AAC​AAA​ACT​GGG​ATG​AGA​AG-3′, Rev 5′-GAC​ATT​CAC​AGG​TCC​TGG​AG-3′), GAPDH (For 5′-ACC​ACA​GTC​CAT​GCC​ATC​AC-3′, Rev 5′-TCC​ACC​ACC​CTG​TTG​CTG​TA-3′).

### Cell viability detection

CCK-8 assay was used to detect the viability of cells cultured in 96-well plate. Then 10 µl CCK-8 solution was added to each well after treatment according to the experimental procedure. After incubation in darkness for 2h, the absorbance of each well was measured with an enzyme marker.

### Cell apoptosis detection

According to the instructions of Annexin V-FITC/PI cell apoptosis detection kit, the cells were re-suspended in the PBS after wash for 3 times. Annexin V-FITC (5 µl) and PI (5 µl) were added and stained for 10 min in dark respectively. Apoptosis rate was detected by flow cytometry.

### Western blot assay

The cells were washed with PBS for 3 times after treatment and collected with 12,000 rpm centrifugation. RIPA was used to lyse cells, then proteins were detected by SDS-PAGE gel electrophoresis and western blot assay. After blocking for 1 h by 5% skim milk, the PVDF membrane was incubated with the primary antibody at 4°C overnight. The membrane was washed 3 times with Tris-buffered saline Tween, and incubated with secondary antibody at room temperature for 2 h. The immunoreactive bands were visualized by ECL chemiluminescence, and the grayscale values of bands were quantified by Image Lab 5.2.1 software. Primary antibodies: MCM2 (1: 2000, ab108935, Abcam), Tau (1: 2000, ab76128, Abcam), p-Tau S404 (1:2000, ab92676, Abcam), GAPDH (1: 2000, ab8245, Abcam). Second antibody: Goat Anti-Rabbit IgG H&L (HRP) (1: 1000, ab6721, Abcam), Rabbit Anti-Mouse IgG H&L (HRP) (1: 1000, ab6728, Abcam).

### Double luciferase reporter gene

The MCM2 3 ′-UTR sequence and the mutated FSP1 3' -UTR sequence was designed and synthesized. The two target gene fragments were cloned into the dual luciferase reporter vector respectively, and the wild-type vector and its mutant vector of the MCM2 3 ′-UTR dual luciferase reporter gene were constructed. The recombinant vector was identified by PCR electrophoresis and gene sequencing to prove that the recombinant vector was constructed successfully. 293T cells were co-transfected with RP11-59J16.2 overexpressed or RP11-59J16.2 negative control using lipofectamineTM2000 transfection reagent. 48 h after transfection, the two recombinant plasmids were analyzed using dual luciferase reporter gene assay (table no. E1960, Promega, Madison, Wisconsin, United States). The dual luciferase reporter gene carrier contains two luciferase genes expressed simultaneously in the same cell, one is firefly luciferase reporter gene, the other is sea renal luciferase reporter gene. Firefly luciferase reporter is the main reporter gene with its fragment cloned into a polyclonal site downstream of the firefly luciferase reporter (Multiple cloning site, MCS). If purpose gene expression were inhibited, the firefly luciferase transcription process would be blocked. Inhibition of the firefly luciferase protein translation would decrease firefly fluorescence value, but not affect the sea renal luciferase expression. So the firefly luciferase activity/sea renal luciferase activity value could determine whether RP11-59J16.2 have direct effect on MCM2 expression.

### Statistical analyses

SPSS 17.0 (Chicago, IL, United States) statistical software was used to analyze the data. The statistical difference between the two groups was calculated by unpaired *t*-test. Benjamini Hochberg FDR (the FDR cutoff was 0.05) was used for multiple-testing correction. One-way analysis of variance (ANOVA) was used to verify the differences between three or more groups. A *p* value < 0.05 was considered as statistically significant difference.

## Results

### Expression profile of mRNAs and long noncoding RNAs in alzheimer’s disease patients and controls

**FIGURE 1:**
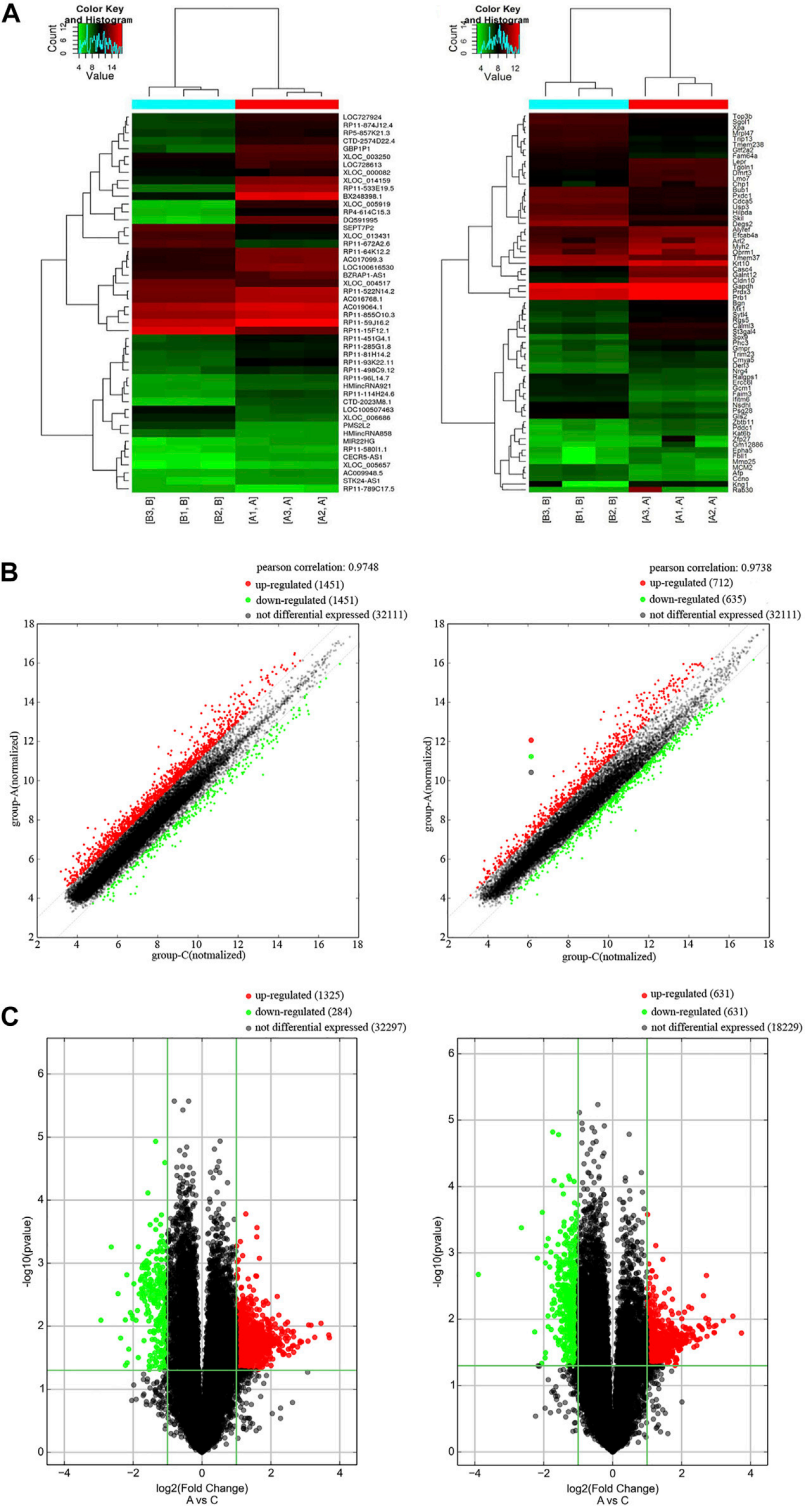
Differentially expression of lncRNAs and mRNAs in AD patients. **(A)** Cluster heat map of the expression profiles of lncRNAs and mRNAs between 3 pairs of AD patients and controls. Red represents up-regulated genes and green represents down-regulated genes. **(B)** Scatter plot of lncRNAs and mRNAs in AD and control. Red represents up-regulated genes and green represents down-regulated genes. **(C)** Volcano plot of mRNA and lncRNA in AD and control. X-axis parallel line: *p* = 0.05; Y-axis parallel line: FC value = 2. Red area, *p* < 0.05, FC ≥ 2 differential genes; Green area, *p* < 0.05, FC ≤ 0.5 differential genes.

We studied the expression profile of mRNAs and lncRNAs of the peripheral blood from AD patients and control group. According to the results, totally 2482 up-regulated lncRNAs, 696 down-regulated lncRNAs, 649 up-regulated mRNAs and 559 down-regulated mRNAs (FC ≥ 2.0 and *p* value ≤ 0.05) were identified. Hierarchical cluster analysis was performed to generate a heat map of differentially expressed lncRNAs and mRNAs (Figure 1A). We used a scatter plot to compare the distribution of differentially expressed lncRNAs and mRNAs (Figure 1B). Based on fold change values and *p* values, a volcano plot was used to show the overall data differential expression distribution of lncRNAs and mRNAs (Figure 1C). In summary, comparing the lncRNAs and mRNAs expression profile of AD patients and control groups, we found significant differences in lncRNAs and mRNAs expression between the two groups.

### Enrichment analyses of the target genes

**FIGURE 2:**
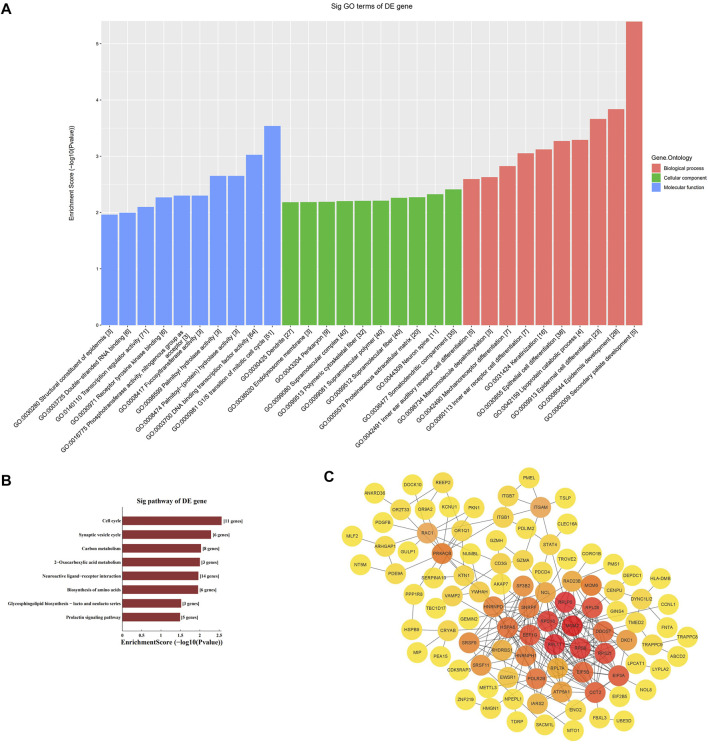
Enrichment biological process of the differentially expression genes. **(A)** GO analysis of differentially expressed mRNA between AD and control group. **(B)** KEGG analysis of differentially expressed mRNA between AD and control group. **(C)** PPI network analysis of mRNA.

We performed GO enrichment and KEGG pathway analysis of the 3178 differentially expressed lncRNAs and 1208 differentially expressed mRNAs to further clarify their functional characteristics (Figure 2). In our research, we found it involved in biological processes, cellular component and molecular functions for example G1/S transition of mitotic cell cycle. KEGG analysis also found that the differentially expressed genes in the AD group and the control group were mainly enriched in signal pathway such as cell circulation, synaptic vesicle cycle, neuroactive receptor-ligand interactions, and Glycosphingolipd biosynthesis (Figures 2A,B).

### Protein-protein interaction network analysis

The STRING online database was used to distinguish the connections between the target genes. A network composed of 99 nodes and 253 edges was obtained, with a confidence score > 0.4 as significant. We used ClusterOne in Cytoscape for gene clustering. The top 50 scoring genes were represented by orange circles, and the color shades were used to correlate significance (Figure 2C).

### RP11-59J16.2 highly expressed in serum of alzheimer’s disease patients and alzheimer’s disease cell models

**FIGURE 3:**
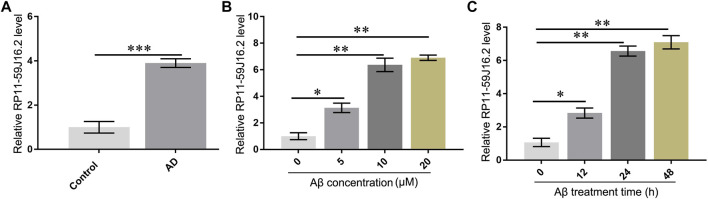
RP11-59J16.2 was overexpressed in serum of AD patients and Aβ treated SH-SY5Y cell model. **(A)** RP11-59J16.2 was highly expressed in serum of AD patients. **(B)** Expression of RP11-59J16.2 was detected in SH-SY5Y after qRT-PCR treatment at different concentrations of Aβ1-42 (0, 5, 10, 20 μM) for 24 h. **(C)**. Expression of RP11-59J16.2 was detected in SH-SY5Y after treatment with 10 μM Aβ1-42 at different times (0, 12, 24, 48 h). * *p* < 0.05; ** *p* < 0.01; *** *p* < 0.001.

According to the results of bioinformatics analysis and qPCR assay, we found that RP11-59J16.2 was up-regulated in the AD patients as showed in the lncRNA differential expression profile. The results of qPCR assay also showed that RP11-59J16.2 was significantly overexpressed in AD patients (Figure 3A). We induced SH-SY5Y cells with Aβ 1–42 (different induce concentration and induce time) to construct the AD cell model to explore the role of RP11-59J16.2 in AD. Expression of RP11-59J16.2 in SH-SY5Y cells increased in a Aβ 1–42 (0, 5, 10, 20 µM) dose- and treat time (0h, 12h, 24 h, 48 h) dependent manner (Figures 3B,C). In the following experiment, 10 μM Aβ treatment for 24 h was used as the condition to induce AD cell model.

### Mini-chromosome maintenance complex component 2 low expressed in alzheimer’s disease cell model

**FIGURE 4:**
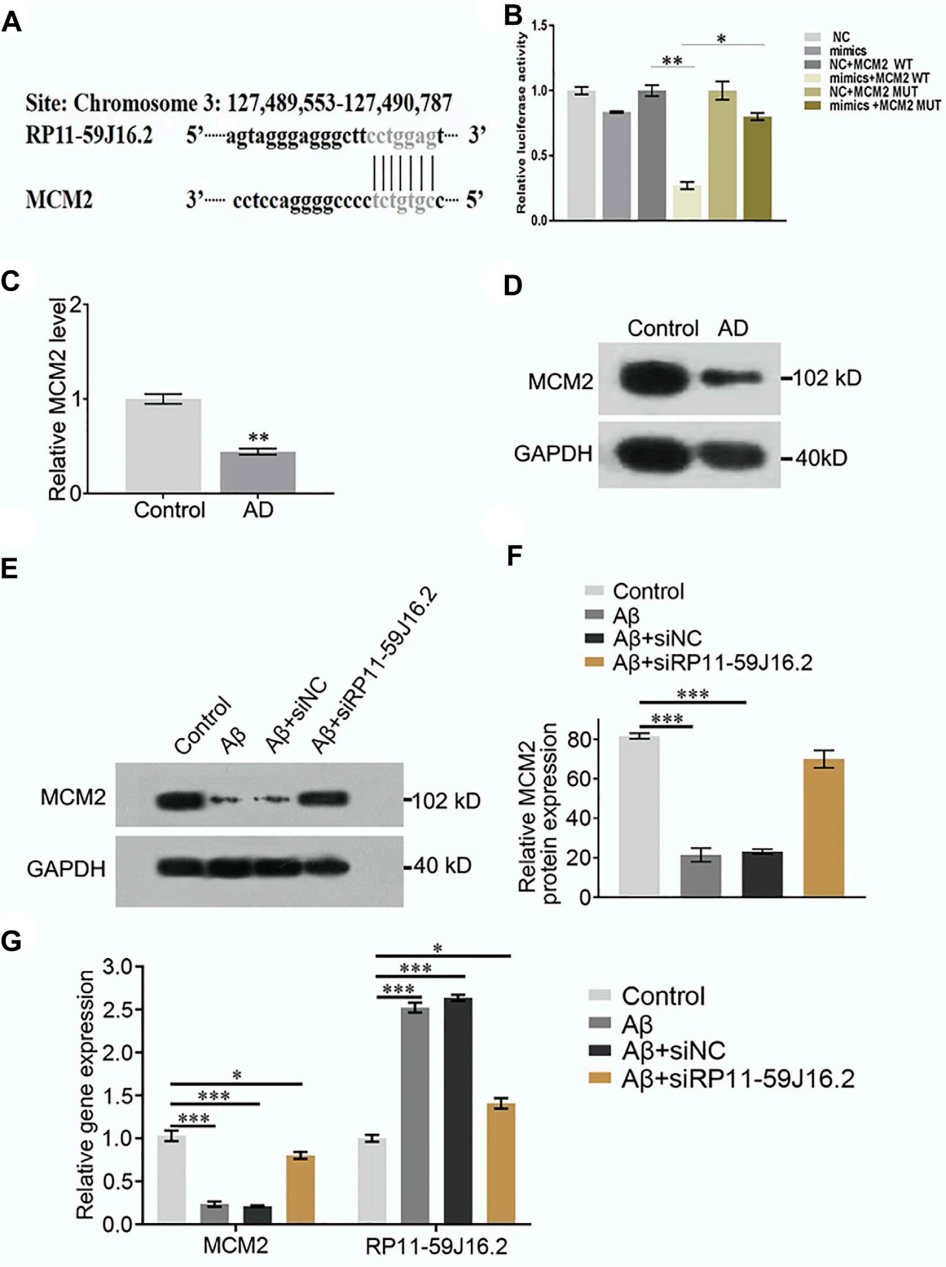
RP11-59J16.2 target regulate the expression of MCM2 which was low expressed AD serum and cell model. **(A)** Bioinformatics analysis of the binding sites of RP11-59J16.2 and MCM2. **(B)** Double luciferase reporter gene assay verified the binding sites of RP11-59J16.2 and MCM2. **(C,D)** Low expression of MCM2 in serum of AD patients by qRT-PCR and Western blot. **(E,F)** MCM2 was low expressed and inhibited by RP11-59J16.2 in AD cell model. **(G)** qRT-PCR confirmed high expression of RP11-59J16.2 and low expression of MCM2 with positive correlation in AD cell model. The experiment was repeated three times in each group, * *p* < 0.05; ** *p* < 0.01, *** *p* < 0.001.

To explore the mechanism of RP11-59J16.2 in the development of AD *in vitro*, we found that MCM2 might be the potential targets of RP11-59J16.2 (Figure 4A) according to the bioinformatics analysis. Lncipedia 5.2 on line data base (https://lncipedia.org/) was used to found the potential lncRNAs which interact with gene. The results of dual luciferase report gene experiment showed that compared to the control group, fluorescein in AD cell model was significantly suppressed after overexpression of RP11-59J16.2 mimics and wild type MCM2. While there was no significant change after overexpression of RP11-59J16.2 mimics and MCM2 mutant (Figure 4B). In addition, qPCR and western blot assay results showed that the expression of MCM2 was significantly decreased in the serum of AD patients (Figures 4C,D). Furthermore, expression of MCM2 reduced after Aβ 1–42 treatment while regained after inhibition of RP11-59J16.2 by co-transfection with si-RP11-59J16.2 *in vitro* (Figures 4E,F). We also confirmed that RP11-59J16.2 could inhibit MCM2 expression in SH-SY5Y cells treated with Aβ 1–42 (Figure 4G).

### RP11-59J16.2 influenced the cell viability and apoptosis of alzheimer’s disease cell model by targeting mini-chromosome maintenance complex component 2

**FIGURE 5:**
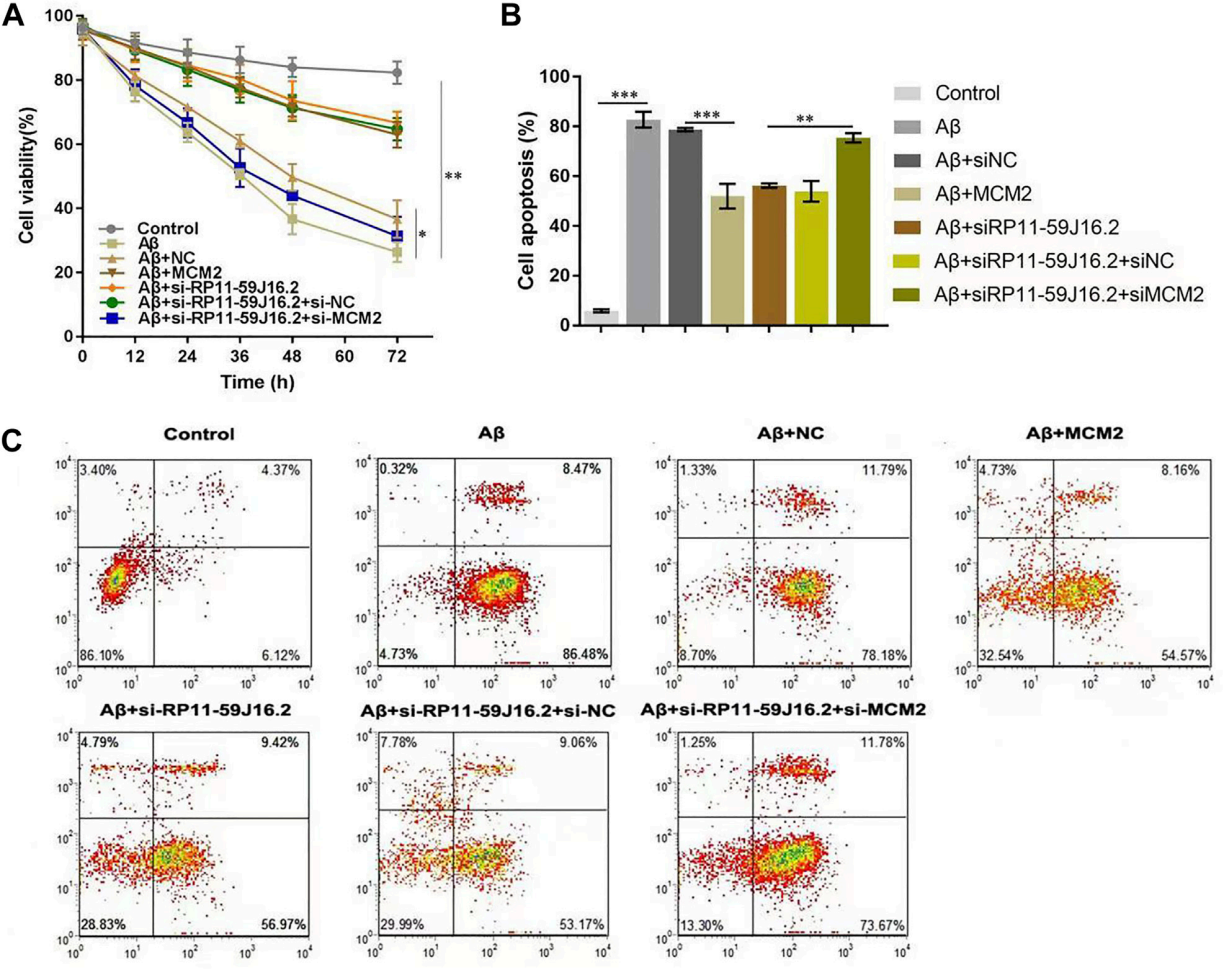
RP11-59J16.2 affect the neuronal damage of SH-SY5Y cells induced by Aβ by targeting MCM2. After treatment with 10 μM Aβ1-42 for 24h, SH-SY5Y cells were transfected with pcDNA-NC, si-RP11-59J16.2, pcDNA-MCM2, si-RP11-59J16.2 + si-NC and si-RP11-59J16.2 + si-MCM2 to detect cell viability **(A)** and apoptosis (B,C). (A) CCK-8 assay was used to detect the changes of cell viability in each group. Cell viability increased significantly in si-RP11-59J16.2 and MCM2 transfection group compared to Aβ treatment group. It reduced significantly after transfection of si-RP11-59J16.2+ si-MCM2. **(B,C)** Flow cytometry was used to explore the effects of RP11-59J16.2 and MCM2 on apoptosis of SH-SY5Y cells. Transfection of si-RP11-59J16.2 or MCM2 significantly reduced the apoptosis of SH-SY5Y cells after Aβ treatment. The cell apoptosis in si-RP11-59J16.2+ si-MCM2 group was significantly increased compared with si-RP11-59J16.2 group. The experiment was repeated three times in each group, * *p* < 0.05; ** *p* < 0.01; *** *p* < 0.001.

To investigate the role of RP11-59J16.2 and MCM2 in the development of AD *in vitro*, SH-SY5Y cells treated with 10 μM Aβ 1–42 were transfected with NC, si-RP11-59J16.2, MCM2, si-RP11-59J16.2 + si-NC, si-RP11-59J16.2 + si-MCM2. CCK-8 assay showed that downregulation of RP11-59J16.2 or overexpression of MCM2 significantly increased cell viability of SH-SY5Y cells, comparing to Aβ treatment group. While the cell viability was reduced significantly in si-RP11-59J16.2 + si-MCM2 group (Figure 5A). Flow cytometry assay was used to study the effect of RP11-59J16.2 and MCM2 on the apoptosis of SH-SY5Y cells. Compare to control group, si-RP11-59J16.2 and overexpression of MCM2 could significantly decrease the apoptosis of SH-SY5Y cells (Figures 5B,C). The cell apoptosis in si-RP11-59J16.2 + si-MCM2 transfected group was significantly increased compared with si-RP11-59J16.2 group. These results indicate that RP11-59J16.2 may influence the cell viability and apoptosis to regulate the neuronal injury in AD by targeting MCM2.

### RP11-59J16.2 increased tau phosphorylation by inhibiting mini-chromosome maintenance complex component 2 in alzheimer’s disease cell model

**FIGURE 6:**
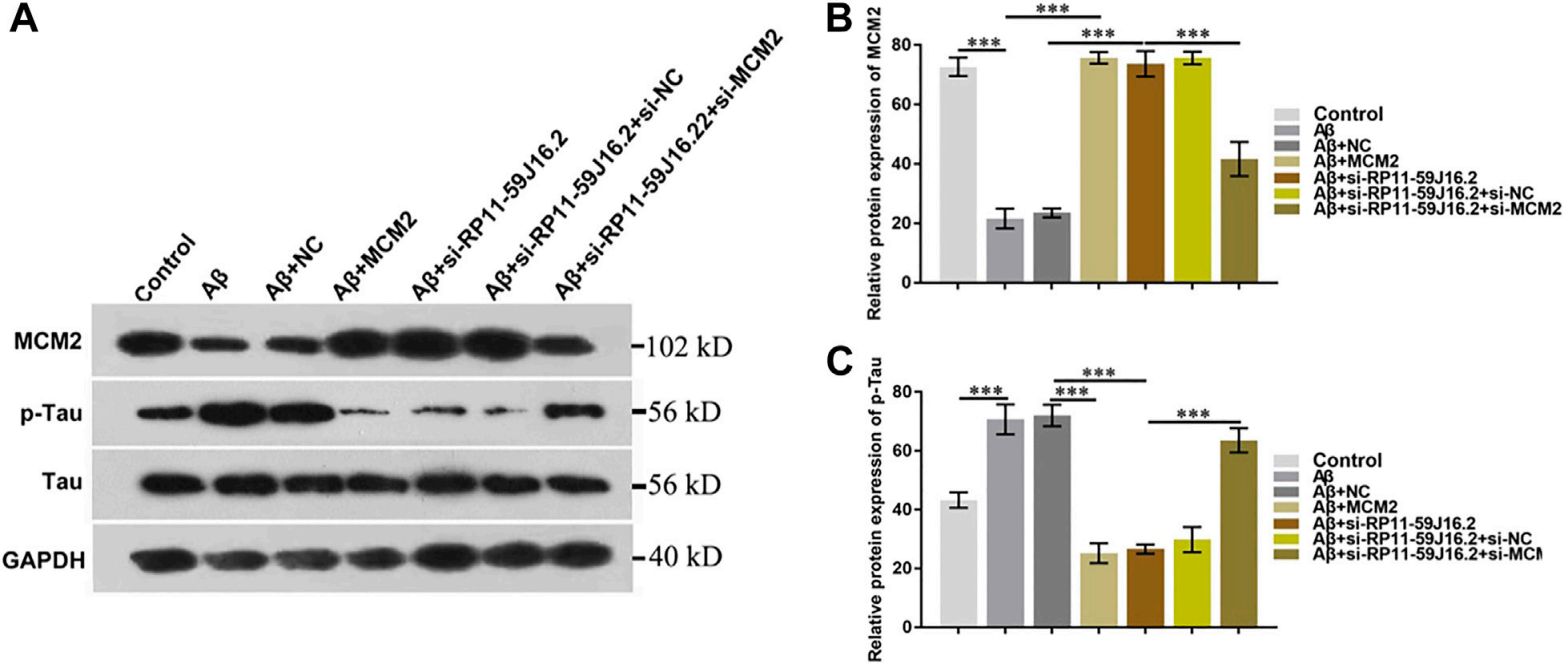
RP11-59J16.2 regulate p-Tau expression by inhibiting MCM2. **(A)** Western blot was used to detect p-Tau and MCM2 expression in each group. p-Tau expression was significantly decreased after transfection with si-RP11-59J16.2 or MCM2. The expression of p-Tau increased significantly in si-RP11-59J16.2 + si-MCM2 transfection group compared with si-RP11-59J16.2 or MCM2 group. The expression of p-Tau and MCM2 was negatively correlated. **(B,C)** Quantitative results of Western blot about the relative expression of MCM2 and p-Tau. The experiment was repeated three times in each group, *** *p* < 0.001.

In order to explore the molecular mechanism of RP11-59J16.2 in the development of AD, western blot assay was used to detect the effect of RP11-59J16.2/MCM2 on the phosphorylation of Tau, as showed in Figure 6A and Figure 6C. The expression of p-Tau increased significantly after treated with Aβ 1–42, but decreased significantly after transfection of si-RP11-59J16.2 or MCM2. Compare to si-RP11-59J16.2 or MCM2 transfection group, the expression of p-Tau increased significantly in si-RP11-59J16.2 + siMCM2 transfection group (Figure 6). mRNA and protein expression of MCM2 significantly reduced after treatment of Aβ 1–42, while its expression increased significantly after inhibition of RP11-59J16.2 in si-RP11-59J16.2 group (Figures 6A,B). These results indicated that RP11-59J16.2 may contribute to AD development through inhibiting of MCM2.

## Discussion

Alzheimer’s disease (AD) is a kind of heterogeneity and multifactor progressive neurodegenerative disease with unclear etiology (Breteler, 2000; Barker et al., 2002; Huang and Mucke, 2012; Bredesen, 2016; Bredesen et al., 2016). Research showed that the incidence rate of AD has increased dramatically in the world in recent years (Fratiglioni et al., 2007; Guerriero et al., 2017). Studying the molecular mechanism of the occurrence and development of AD could provide a theoretical basis for AD prevention and treatment. Long non-coding RNA (lncRNA) could regulate gene expression in transcription, post-transcription, epigenetics and other aspects, and further regulate cell proliferation, differentiation, apoptosis and other life processes, thus it has been found closely related to the occurrence and development of diseases (Wei et al., 2017; Wu et al., 2018). Several lncRNAs may be involved in the pathological process of AD through Aβ deposition, synaptic remodeling and memory formation. Huaying et al. (2020) reported that 5 lncRNAs for predicting the prognosis of AD based on competing endogenous RNA networks. Zhang et al. (2021) found that silenced lncRNA H19 and up-regulated microRNA-129 could accelerate viability and restrain apoptosis of PC12 cells induced by Aβ in a cellular model of AD. It is of great clinical value to discover and study new lncRNAs that regulate the occurrence and development of AD.

In this study, the differential expression profile of mRNA and lncRNA in the peripheral blood from AD patients was analyzed. Results showed that totally 2482 lncRNAs were up-regulated, 696 lncRNAs were down-regulated, 649 mRNAs were up-regulated and 559 mRNAs were down-regulated. Subsequently, GO analysis and KEGG analysis were performed on the differentially expressed mRNAs. GO analysis results showed that differentially expressed mRNAs were involved in many biological processes, cell composition and molecular functions, mainly enriched in G1/S transition of mitotic cell cycle, suggesting that G1/S transition of mitotic cell cycle may be involved in the occurrence of Alzheimer’s disease. KEGG analysis also found that the differentially expressed genes in AD patients were mainly in the signal pathways including cell circulation, synaptic vesicle cycle, neuroactive receptor-ligand interactions, and Glycosphingolipd biosynthesis. This is consistent with previous studies that synapses are essential for cognitive function and that synaptic loss is a stable pathological phenomenon in AD (Selkoe, 2002; Scheff et al., 2014). Some studies have confirmed that synaptic loss of cortex and limbic system is closely related to AD cognitive dysfunction (DeKosky and Scheff, 1990; Terry et al., 1991). In addition, chronic neuroinflammatory changes are also an important neuropathological process in AD. The findings suggest that activation of microglia and astrocytes is involved in the progression of AD, although inflammatory glial responses are thought to be secondary to neuronal death or dysfunction (Lampron et al., 2013). Sphingomyelin and its metabolites play important roles in several cellular processes and signaling pathway, including neuroinflammation, which in turn contribute to the development of AD (de Wit et al., 2020). Moreover, astrocytes regulate synaptic remodeling by releasing synaptic plasticity and neuronal excitability of neuroactive substances such as glial transmitters such as ATP, glutamate, D-serine and GABA, which bind to receptors in the pre - and post-synaptic membranes (Nanclares et al., 2021). Microglia express pattern recognition receptors (PRRs) that bind to different types of Aβ (Venegas and Heneka, 2017). *In vitro* experiments confirmed that Aβ activates microglia by binding PRRs, including receptors for advanced glycation end products (RAGE), TLRs and scavenger receptors (Sarlus and Heneka, 2017). Production of pro-inflammatory factors would increase microglial phagocytosis by binding of these pathogen-associated molecular patterns (DAMPs or PAMPs) to PRRs (Pan et al., 2011). All above suggest that neuro-activated receptors and ligands are involved in the pathological mechanism of AD.

Recent studies have found that neuronal cell cycle re-entry is involved in the development of AD (McShea et al., 2007; Neve and McPhie, 2007). Cell cycle regulators are involved in neuronal migration, neuronal maturation and synaptic plasticity in adult neurons (Lim et al., 2013). Cell cycle markers are upregulated and reactivated in response to acute injury such as neurotrophic factor deprivation, DNA damage, oxidative stress, and excitatory toxicity, and lead to the death of sympathetic and cortical neurons at G1/S prior to DNA synthesis (Liu and Greene, 2001; Becker and Bonni, 2004). This suggests that in some specific neuronal phenotypes, many signaling pathways triggered by different environmental conditions may cause cell cycle activation and cell death. It is consistent with our results of GO analysis and KEGG analysis which showed that differentially expressed genes are mainly enriched in G1/S transition of mitotic cell cycle and cell cycle signaling pathway. Therefore, our results also prove the involvement of the cell cycle in the pathogenesis of AD.

Among down-regulated genes, MCM2 was an important gene which effect the development of AD by promoting neuronal injury repair. MCM2 encodes the microchromosome maintenance complex (MCM), a key factor in the initiation of genome replication in eukaryotes (Lei et al., 1997). Previous studies have demonstrated that MCM2 is expressed in aging hippocampus (Wharton et al., 2005). MCM2 has been reported to play an important role in the development of AD. Wharton et al. (2005) reported that the expression of the MCM2 was associated with the burden of Alzheimer-type pathology. Bonda et al. (2009) proved that phosphorylated MCM2 (pMCM2) was markedly associated with neurofibrillary tangles, neuropil threads, and dystrophic neurites in AD. The study of Mezache et al. (2015) showed that as a marker of the neuroprogenitor cells, the expression of MCM2 was significantly reduced in the Alzheimer sections that contained the hyperphosphorylated Tau.

Lncipedia 5.2 on line data base (https://lncipedia.org/) was used to found the potential lncRNAs which interact with MCM2 gene. Results showed that up-regulated lncRNA RP11-59J16.2 in AD patients was the potential lncRNA. RP11-59J16.2 was significantly overexpressed in serum of AD patients, while MCM2 was significantly reduced. The result was consistent in AD cell model (SH-SY5Y cells which treated with 10 μM Aβ 1–42). The relationship between AD pathological mechanism and RP11-59J16.2/MCM2 is unclear. Subsequent results showed that MCM2 was the direct target of RP11-59J16.2 and RP11-59J16.2 could regulate the expression of MCM2. Our results suggested that RP11-59J16.2 might affect cell viability and apoptosis in AD cell model by inhibiting MCM2, which consist to the idea that cell cycle re-entry may cause neuronal death by apoptosis (Yang et al., 2003). Previous result (Bonda et al., 2009) provided further evidence for cell cycle aberrations in AD and they also found that cytoplasmic localization of pMCM2 may explain resultant cell cycle stasis and consequent neuronal degeneration. Combined with our result, it proved that cell cycle re-entry would be involved in the pathogenesis of AD from bioinformatics analysis as mentioned before and illustrated that RP11-59J16.2 might contribute to AD development in this pathway by targeting MCM2. Then the molecular mechanism of RP11-59J16.2 in the development of AD was further investigated. The expression of p-Tau increased decreased significantly with the transfection of si-RP11-59J16.2 or MCM2 transfection. After inhibition of MCM2 by co-transfection with si-MCM2, the expression of p-Tau increased significantly. Those results showed that RP11-59J16.2 could increase Tau phosphorylation through inhibiting MCM2 expression in SH-SY5Y cells treated by Aβ.

Although the present study provided a noval lncRNA-- RP11-59J16.2 probably related to the development of AD by affecting cell apoptosis and Tau phosphorylation through regulation of MCM2, it still had some limitations. First, the sample size for microarray analysis was small, which may affect the validity of sequencing results. Secondly, the microarray analysis samples we collected were serum samples of patients, which may be affected by many other external factors. Microarray analysis of cerebrospinal fluid would improve the specificity of the results. However, patients and controls rarely cooperate to complete the lumbar puncture invasive examination clinically. Finally, no *in vivo* experiments were performed. Future studies need to explore the concrete interaction between lncRNA-- RP11-59J16.2 and MCM2, and the downstream mechanism of MCM2 on Tau phosphorylation, cell apoptosis and cell-cycle activation in AD.

## Conclusion

In this study, the differential expression profile of mRNA and lncRNA in the peripheral blood of AD patients was analyzed. Together our data suggest that RP11-59J16.2 may contribute to the development of AD by targeting MCM2 on apoptosis and Tau phosphorylation. Our results may illustrate that RP11-59J16.2 might be a potential target molecule for the prediction, diagnosis and treatment of AD. Still, further studies about its molecular mechanism should be explored *in vivo*.

## Data Availability

The datasets presented in this study can be found in online repositories. The names of the repository/repositories and accession number(s) can be found in the article/Supplementary Material

## Abbreviations

AD, Alzheimer’s disease; GO, Gene Ontology; HAMD, Hamilton Depression Scale; KEGG, Kyoto Encyclopedia of Genes and Genomes; lncRNA, long non-coding RNA; MCM2, Mini-chromosome maintenance complex component 2; NINCDS-ADRDA, National Institute of Neurological and Communicative Disorders and Stroke/Alzheimer’s disease Criteria; PPI, Protein-Protein Interaction; SH-SY5Y, Human neuroblastoma cell line.
