# Supplementary figures and images for: LncRNA RP11-59J16.2 aggravates apoptosis and increases tau phosphorylation by targeting MCM2 in AD
Source: Front Genet. 2022 Aug 26;13:824495. doi: 10.3389/fgene.2022.824495 (PMC9459667; doi:10.3389/fgene.2022.824495)

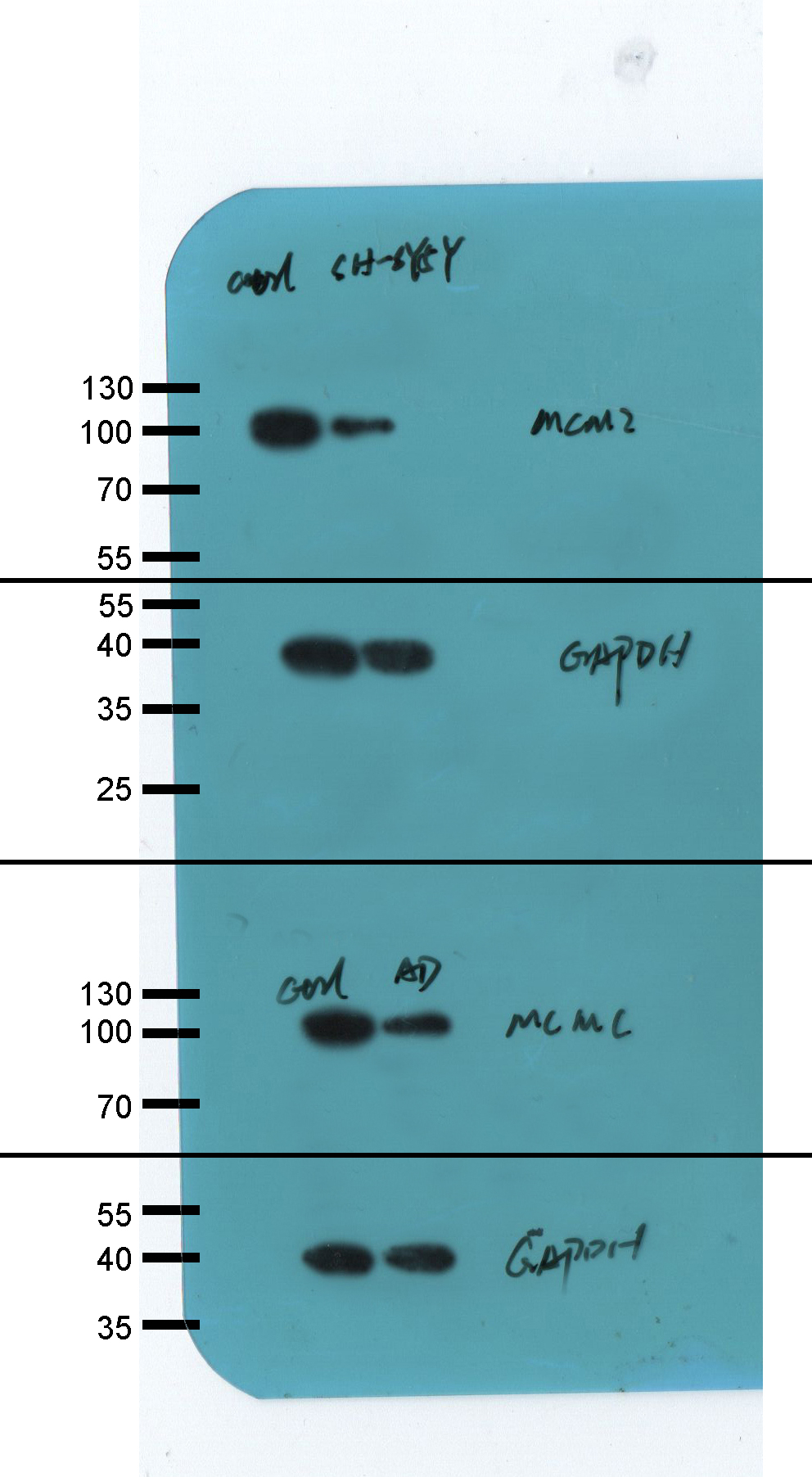

Supplement: Supplementary file 5 [file Image1.JPEG]

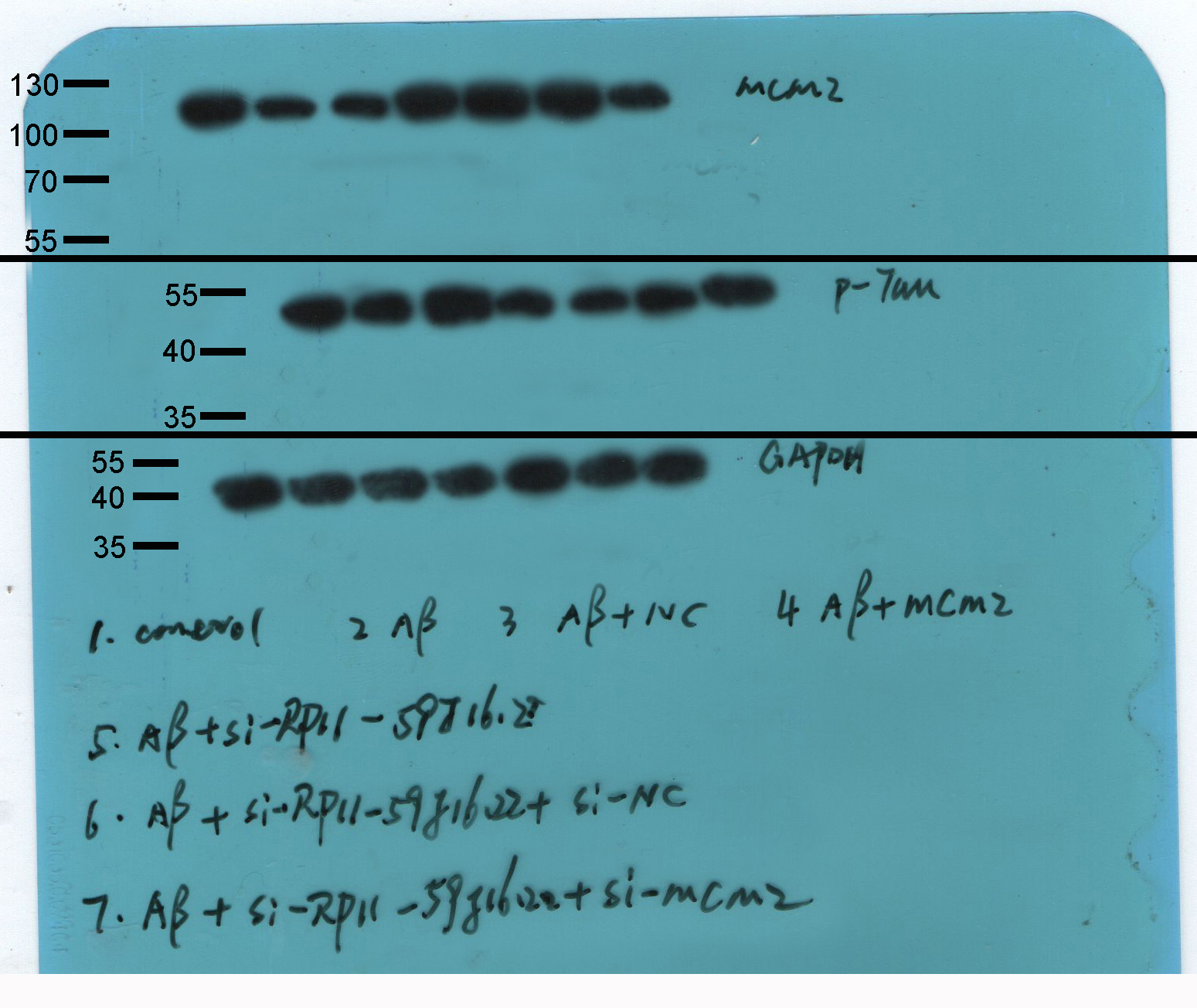

Supplement: Supplementary file 6 [file Image2.JPEG]
